# Sulphamoylated Estradiol Analogue Induces Reactive Oxygen Species Generation to Exert Its Antiproliferative Activity in Breast Cancer Cell Lines

**DOI:** 10.3390/molecules25184337

**Published:** 2020-09-22

**Authors:** Maphuti T. Lebelo, Anna M. Joubert, Michelle H. Visagie

**Affiliations:** Department of Physiology, University of Pretoria, Private Bag X323, Gezina, Pretoria 0031, South Africa; tebogo_lebelo@yahoo.com (M.T.L.); annie.joubert@up.ac.za (A.M.J.)

**Keywords:** sulphamoylated, ESE-one, tiron, trolox, DMTU, antiproliferation

## Abstract

2-Methoxyestradiol (2ME), a 17β-estradiol metabolite, exerts anticancer properties in vitro and in vivo. To address 2ME’s low bioavailability, research led to the in silico design of sulphamoylated 2ME analogues. However, the role of oxidative stress induced in the activity exerted by sulphamoylated compounds remains elusive. In the current study, the influence of 2-Ethyl-17-oxoestra-1,3,5(10)-trien-3-yl sulphamate (ESE-one) on reactive oxygen species (ROS) induction and its effect on cell proliferation, as well as morphology, were assessed in breast tumorigenic cells (MCF-7 and MDA-MB-231). Fluorescent microscopy showed that sulphamoylated estradiol analogues induced hydrogen peroxide and superoxide anion, correlating with decreased cell growth demonstrated by spectrophotometry data. ESE-one exposure resulted in antiproliferation which was repressed by tiron (superoxide inhibitor), trolox (peroxyl inhibitor) and *N*,*N*′-dimethylthiourea (DMTU) (hydrogen peroxide inhibitor). Morphological studies demonstrated that tiron, trolox and DMTU significantly decreased the number of rounded cells and shrunken cells in MCF-7 and MDA-MB-231 cells induced by ESE-one. This in vitro study suggests that ESE-one induces growth inhibition and cell rounding by production of superoxide anion, peroxyl radical and hydrogen peroxide. Identification of these biological changes in cancer cells caused by sulphamoylated compounds hugely contributes towards improvement of anticancer strategies and the ROS-dependent cell death pathways in tumorigenic breast cells.

## 1. Introduction

2-Methoxyestradiol (2ME), a 17-β estradiol metabolite, exhibits antiangiogenic and antitumor activity [1]. Furthermore, 2ME is destructive to the tubulin structure of cells regardless of the estrogen receptor status and induces apoptosis. Despite the desired effects that 2ME has on tumorigenic cells, it has low bioavailability and is easily degraded. This led to the in silico design of several sulphamoylated and non-sulphamoylated estradiol compounds with improved bioavailability [2] (Table 1).

Several estradiol sulphamoylated analogues including 2-methoxyestradiol-bis-sulphamate, (8*R*,13*S*,14*S*,17*S*)-2-ethyl-13-methyl-7,8,9,11,12,13,14,15,16,17-decahydro-6H-cyclopenta[a]phenanthrane-3,17-diyl bis(sulphamate) (EMBS), 2-ethyl-17-hydroxyestra-1(10),2,4,16-tetreane-3-yl sulfamate (ESE-15-ol) and 2-ethyl-17-hydroxyestra-1(10),2,4-trien-3-yl sulfamate (ESE-ol) exert antiproliferative, antimitotic and apoptotic activity in tumorigenic cell lines including breast tumorigenic cell lines (MCF-7, MDA-MB-231) and an esophageal tumorigenic cell line (SNO) [2,3,4,5,6,7]. The addition of N-acetyl cysteine (NAC) abrogated antiproliferative activity, cell cycle arrest and cell death suggesting that ROS production is essential for the ability of EMBS to induce cell death. However, the specific ROS-dependent signaling cascade induced by EMBS including the relevant ROS involved remains unknown [8].

ROS are oxygen species possessing an unpaired electron which are highly reactive [9]. ROS include, among others, singlet oxygen, superoxide radical, perhydroxyl radical, hydrogen peroxide and hydroxyl radical, and play a key role in apoptosis induction [10,11,12,13]. However, an excessive quantity of ROS is known to induce cell death including via the apoptotic pathways [8,9,10]. The identification of aberrant ROS modulated by sulphamoylated estradiol antimitotic compounds in cancer cells therefore contributes towards the understanding of oxidative-stress-dependent signaling and improvement of anticancer strategies.

## 2. Results

### 2.1. Sulphamoylated Compounds Induce Hydrogen Peroxide and Superoxide Anion

**Figure 1:**
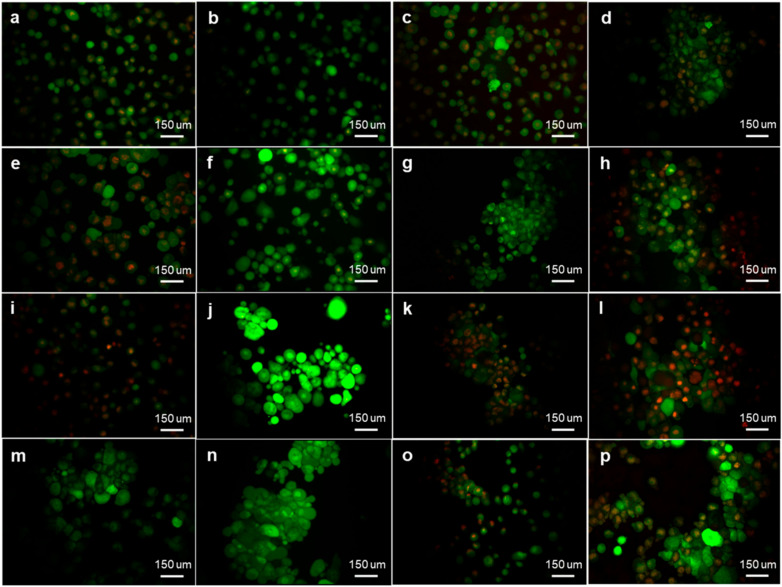
Fluorescent micrographs of MCF-7 and MDA-MB-231 cells exposed to sulphamoylated and non-sulphamoylated compounds. Sulphamoylated compounds induced ROS production in both MCF-7 and MDA-MB-231 cells compared to their non-sulphamoylated counterparts. (**a**) MCF-7 cells propagated in growth medium, (**b**) vehicle-treated MCF-7 cells, (**c**) MDA-MB-231 cells propagated in growth medium, (**d**) vehicle-treated MDA-MB-231 cells, (**e**) EE-15-ol (non-sulphamoylated) treated MCF-7 cells, (**f**) ESE-15-ol (sulphamoylated)-treated MCF-7 cells, (**g**) EE-15-ol (non-sulphamoylated)-treated MDA-MB-231 cells, (**h**) ESE-15-ol (sulphamoylated)-treated MDA-MB-231 cells, (**i**) EE-one (non-sulphamoylated)-treated MCF-7 cells, (**j**) ESE-one (sulphamoylated)-treated MCF-7 cells, (**k**) EE-one (non-sulphamoylated)-treated MDA-MB-231 cells, (**l**) ESE-one (sulphamoylated)-treated MDA-MB-231 cells, (**m**) 2E-diol (non-sulphamoylated)-treated MCF-7 cells, (**n**) ESE-ol (sulphamoylated)-treated MCF-7 cells, (**o**) 2E-diol (non-sulphamoylated)-treated MDA-MB-231 cells, (**p**) ESE-ol (sulphamoylated)-treated MDA-MB-231 cells. Images were captured at 20X magnification.

**Figure 2:**
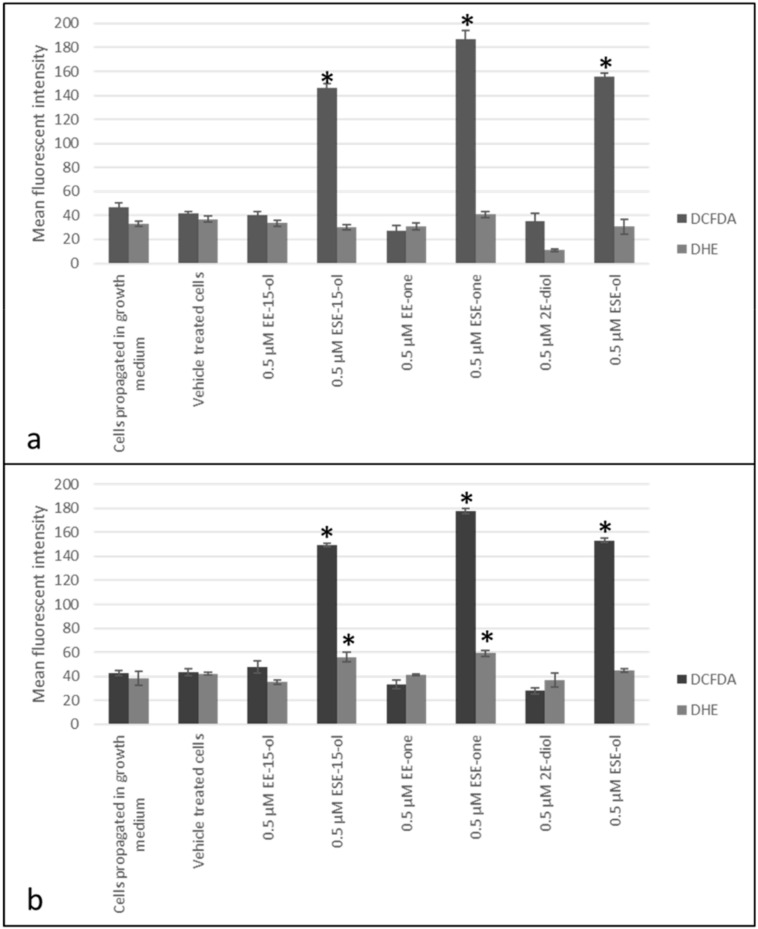
MCF-7 and MDA-MB-231 graphs demonstrating the mean fluorescent intensity following exposure to sulphamoylated and non-sulphamoylated compounds. Sulphamoylated compounds induced superoxide anion and hydrogen peroxide production in both MCF-7 and MDA-MB-231 cells compared to the non-sulphamoylated compounds. (**a**) MCF-7 cells, (**b**) MDA-MB-231 cells. Asterisk (*) represents *p*-value (*p* < 0.05) compared to vehicle-treated cells.

Fluorescent microscopy studies were conducted to evaluate ROS (superoxide anion and hydrogen peroxide) production induced by sulphamoylated compounds utilizing dihydroethidine (DHE) and 2,7-dichlorofluoresceindiacetate (DCFDA) (Figure 1 and Figure 2). ESE-15-ol exposure induced hydrogen peroxide production with a mean florescent intensity of 146 and 149, and a mean fluorescent intensity of 30 and 56 for superoxide anion production in MCF-7 and MDA-MB-231 cells, respectively (Figure 1f,h). 2-Vinylestra-1(10),2,4,16-tetraene-3,17-diol (EE-15-ol) exposure demonstrated a mean fluorescent intensity of 40 and 48 for hydrogen peroxide, and a mean fluorescent intensity of 33 and 35 for superoxide anion production in MCF-7 and MDA-MB-231 cells, respectively (Figure 1e,g). 2-Ethyl-17-oxoestra-1,3,5(10)-trien-3-yl sulphamate (ESE-one) exposure resulted in a mean fluorescent intensity of 187 and 178 for hydrogen peroxide production and a mean fluorescent intensity of 41 and 59 for superoxide anion production in MCF-7 and MDA-MB-231 cells, respectively (Figure 1j,l). 2-Ethyl-3-hydroxyestra-1(10),2,4-trien-17-one (EE-one) exposure resulted in a mean fluorescent intensity of 27 and 33 for hydrogen peroxide production and a mean fluorescent intensity of 31 and 41 for superoxide anion production in MCF-7 and MDA-MB-231 cells, respectively (Figure 1i,k). ESE-ol exposure resulted in a mean fluorescent intensity of 156 and 153 for hydrogen peroxide production and a mean fluorescent intensity of 31 and 45 for superoxide anion production in MCF-7 and MDA-MB-231 cells, respectively (Figure 1n,p). 2E-diol exposure demonstrated a mean fluorescent intensity of 35 and 28 for hydrogen peroxide and a mean fluorescent intensity of 11 and 37 for superoxide anion production in MCF-7 and MDA-MB-231 cells, respectively (Figure 1m,o). Fluorescent microscopy data revealed that sulphamoylated compounds induce ROS production in both MCF-7 (Figure 2a) and MDA-MB-231 cells (Figure 2b) compared to the non-sulphamoylated compounds. Furthermore, sulphamoylated compounds increased hydrogen peroxide production in MCF-7 cells compared to MDA-MB-231 cells. An increase in superoxide anion production in MDA-MB-231 cells was also observed compared to MCF-7 cells. Thus, exposure to all the sulphamoylated compounds resulted in induction of superoxide anion and hydrogen peroxide compared to the vehicle-treated cells. Exposure to the non-sulphamoylated compounds, however, induced less superoxide anion and hydrogen peroxide suggesting that ROS production is more prominently induced by sulphamoylated compounds.

### 2.2. Decline in Cell Growth Owed to Sulphamoylated Compounds

**Figure 3:**
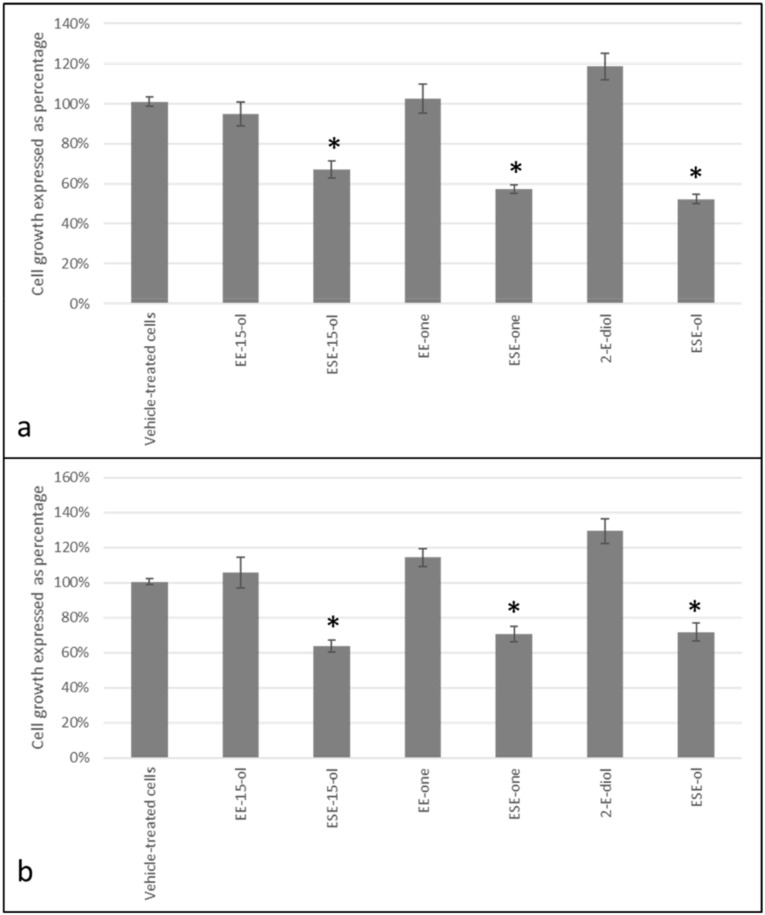
Graph of MCF-7 and MDA-MB231 cells illustrating effect on proliferation after exposure to sulphamoylated and non-sulphamoylated compounds. Non-sulphamoylated compounds exerted no significant inhibiting effect on cell growth in MCF-7 cell inhibition whereas sulphamoylated compounds demonstrated at least 28% cell inhibition in both cell lines. Non-sulphamoylated compounds had an opposite effect and caused cell growth demonstrated by EE-one and 2-E-diol. (**a**) MCF-7 cells, (**b**) MDA-MB-231 cells. Asterisk (*) represents *p*-value (*p* < 0.05) compared to cells exposed to non-sulphamoylated compounds.

MCF-7 and MDA-MB-231 cell lines were exposed to three types of sulphamoylated compounds (ESE-15-ol, ESE-one and ESE-ol) and their non-sulphamoylated (EE-15-ol, EE-one and 2-ethylestra-1(10),2,4-triene-3,17-diol (2-E-diol) counterparts in order to determine the effect of sulphamoylated compounds on tumorigenic cell lines in comparison to non-sulphamoylated compounds. Cells were exposed to sulphamoylated and non-sulphamoylated compounds for 24 h at a concentration of 0.5 µM. Cells exposed to EE-15-ol exhibited 95% cell growth in the MCF-7 cell line (Figure 3a) and 106% cell growth in the MDA-MB-231 cell line (Figure 3b) compared to those exposed to its sulphamoylated counterpart (ESE-15-ol) which resulted in only 67% cell growth in the MCF-7 cell line and 64% cell growth in the MDA-MB-231 cell line. EE-one exposure resulted in 102% and 114% cell growth in MCF-7 and MDA-MB-231 cell lines, respectively, whereas ESE-one exposure demonstrated 57% cell growth in the MCF-7 cell line and 71% growth in the MDA-MB-231 cell line. 2-E-diol exposure resulted in 119% and 130% cell growth in MCF-7 and MDA-MB-231 cell lines compared to 52% and 72% growth, respectively (Figure 3a,b). Crystal violet studies demonstrated that the compounds owning a sulphamate moiety indeed have a significant inhibitory effect on cell growth as they exhibited more prominent cell growth inhibition compared to their non-sulphamoylated counterparts which had the opposite effect by inducing cell growth.

ESE-one was chosen as a representative for the sulphamoylated compounds and was thus used in subsequent experiments.

### 2.3. ROS Scavengers Oppose the Antiproliferative Effects of Sulphamoylated Compounds (ESE-One)

Cell growth studies were done using 0.5 µM ESE-one in the presence or absence of ROS inhibitors. These inhibitors include mannitol which inhibits hydroxyl radical, sodium azide which inhibits oxygen singlet, 2-(4-carboxyphenyl)-4,4,5,5-tetramethylimidazoline-1-oxyl-3-oxide (Carboxy-PTIO), which inhibits nitric oxide, tiron which inhibits superoxide anion, *N*,*N*′-dimethylthiourea (DMTU) which inhibits hydrogen peroxide and trolox which inhibits perhydroxyl radical.

**Figure 4:**
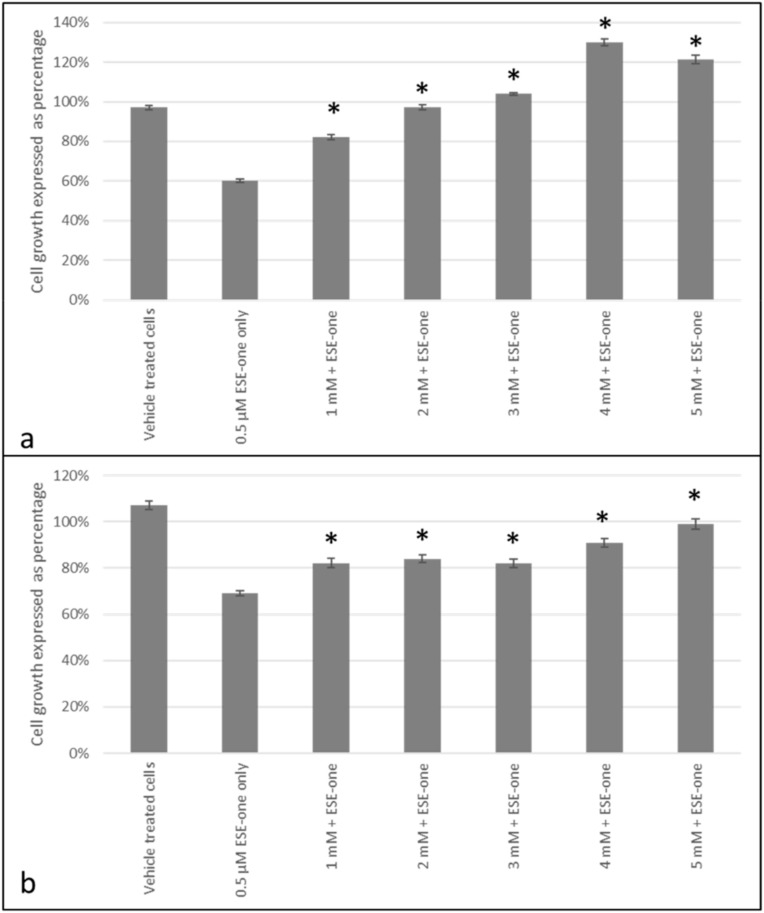
Cell growth inhibition graphs of MCF-7 and MDA-MB-231 cell lines exposed to ESE-one in the presence or absence of tiron (superoxide anion inhibitor). Tiron exposure to MCF-7 and MDA-MB-231 cells significantly opposed the antiproliferative effect of ESE-one. The growth inhibitory effect of ESE-one was demolished at 3 mM in MCF-7 cells and 5 mM in MDA-MB-231 cells. (**a**) MCF-7 cells, (**b**) MDA-MB-231 cells. Asterisk (*) represents a *p* value of <0.05 compared to ESE-one treated cells.

Tiron, an inhibitor of superoxide anion, was used to determine if the growth inhibitory effect of ESE-one is dependent on superoxide. Co-exposure to tiron resulted in a significant restoration of cell growth to 82% (1 mM), 97% (2 mM), 104% (3 mM), 130% (4 mM) and 121% (5 mM) compared to cells exposed to ESE-one only which demonstrated 66% cell growth in MCF-7 cells (Figure 4a). Tiron exposure significantly increased cell growth at just 1 mM and completely obliterated ESE-one’s growth inhibitory effect at 3 mM concentration in MCF-7 cells. Furthermore, tiron exposure in MDA-MB-231 cells restored cell growth to 82% (1 mM), 84% (2 mM), 82% (3 mM), 91% (4 mM) and 99% (5 mM) compared to ESE-one only exposure which resulted in 69% cell growth (Figure 4b). Furthermore, tiron also demonstrates a significant opposing effect to the antiproliferative effect exerted by ESE-one in MDA-MB-231 cells. This suggests that the superoxide anion production is induced by ESE-one exposure culminating in decreased cell growth. A concentration of 5 mM was chosen to continue for subsequent experiments since it was the only concentration which completely obliterated the antiproliferative effects of ESE-one in both MCF-7 and MDA-MB-231 cell lines.

**Figure 5:**
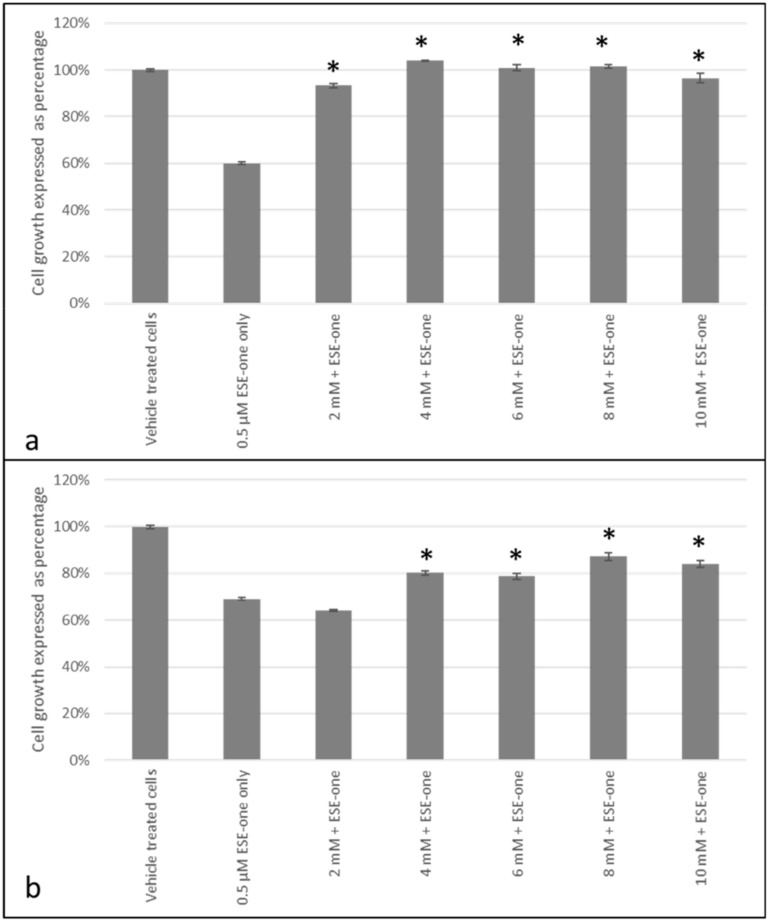
Cell growth inhibition graphs of MCF-7 and MDA-MB-231 cells exposed to ESE-one in combination with DMTU (*N*,*N*′-dimethylthiourea, hydrogen peroxide inhibitor). DMTU exposure to MCF-7 and MDA-MB-231 cells significantly opposed the antiproliferative effect of ESE-one in both cell lines. The antiproliferative effect of ESE-one was demolished at 4 mM in MCF-7 cells and was significantly inhibited at 8 mM in MDA-MB-231 cells. (**a**) MCF-7 cells, (**b**) MDA-MB-231 cells. Asterisk (*) represents *p*-value (*p* < 0.05) compared to ESE-one treated cells.

DMTU, an inhibitor of hydrogen peroxide, was used to evaluate if antiproliferative activity induced by ESE-one in MCF-7 and MDA-MB-231 cell lines is dependent on the production of hydrogen peroxide. Co-exposure to DMTU restored cell growth to 93% (2 mM), 104% (4 mM), 101% (6 mM), 102% (8 mM) and 96% (10 mM) compared to 60% cell growth induced by ESE-one exposure in MCF-7 cells (Figure 5a). These results demonstrate that DMTU inhibits the antiproliferative effect exerted by ESE-one from a concentration of 2 mM, suggesting that hydrogen peroxide plays an essential role in the antiproliferative effect induced by ESE-one. DMTU exposure to MDA-MB-231 cells restored cell growth to 64% (2 mM), 80% (4 mM), 79% (6 mM), 87% (8 mM) and 84% (10 mM) compared to 69% cell growth induced by ESE-one (Figure 5b). DMTU exposure significantly increases cell growth in MDA-MB-231 exposed cells at 8 mM. However, cell growth was only partially restored by DMTU in the MDA-MB-231 cell line.

**Figure 6:**
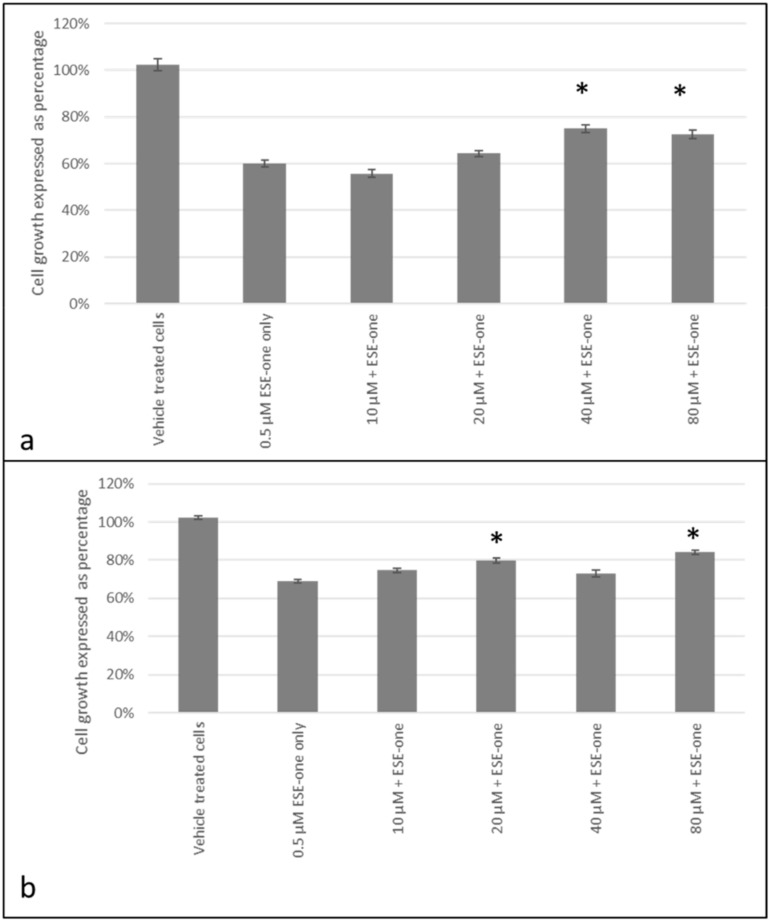
Cell growth inhibition graphs demonstrating MCF-7 and MDA-MB-231 cells exposed to ESE-one in combination with trolox (peroxyl radical inhibitor). Trolox exposure to MCF-7 and MDA-MB-231 cells partially opposed the antiproliferative effect of ESE-one in both cell lines. The antiproliferative effect of ESE-one was significantly countered at 40 µM and 80 µM in MCF-7 cells and 80 µM in MDA-MB-231 cells. (**a**) MCF-7 cells, (**b**) MDA-MB-231 cells. Asterisk (*) represents *p*-value (*p* < 0.05) compared to ESE-one treated cells.

**Figure 7:**
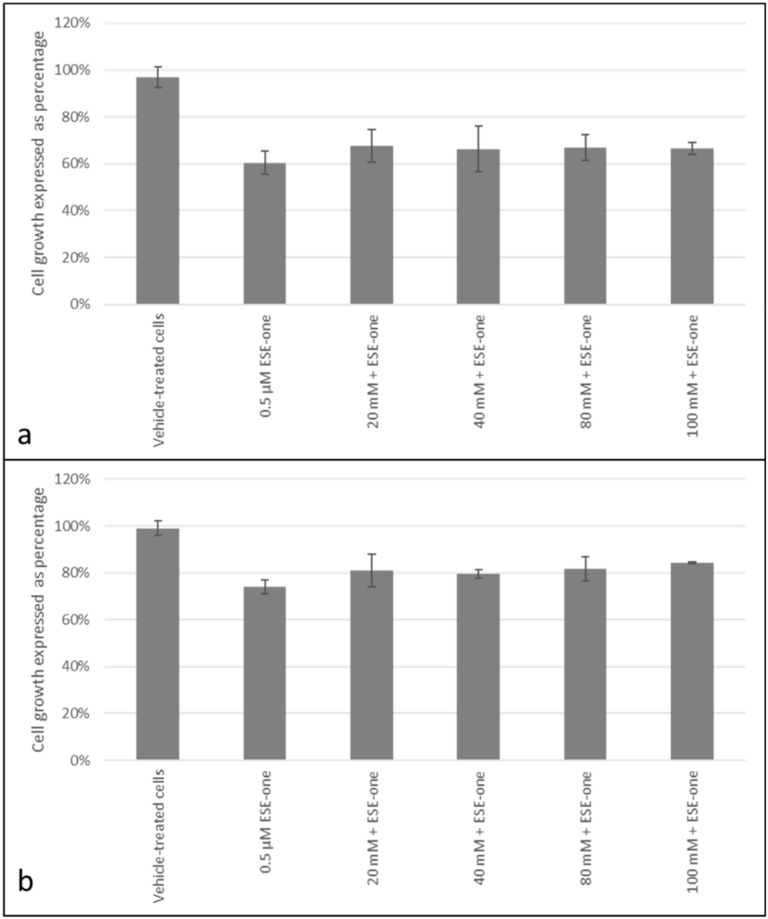
Cell growth inhibition graphs of MCF-7 and MDA-MB-231 cells exposed to ESE-one in combination with mannitol (hydroxyl radical inhibitor). Mannitol exposure to MCF-7 and MDA-MB-231 cells did not significantly oppose the antiproliferative effect of ESE-one in both cell lines. (**a**) MCF-7 cells, (**b**) MDA-MB-231 cells. No significant differences were observed in cell growth when compared to ESE-one treated cells (*p* > 0.05).

Trolox, a peroxyl radical inhibitor, was used to determine if the antiproliferative effects induced by ESE-one are dependent on production of peroxyl radical. Co-exposure to trolox and ESE-one resulted in 56% (10 µM), 64% (20 µM), 75% (40 µM) and 72% (80 µM) compared to cells exposed to ESE-one only (60%) in MCF-7 cells (Figure 6a). Thus, trolox significantly opposed the antiproliferative effect of ESE-one at in a dose-dependent manner at 40 µM and 80 µM. In MDA-MB-231 cells, trolox exposure restored cell growth to 75% (10 µM), 80% (20 µM), 73% (40 µM) and 84% (80 µM) compared to ESE-one only exposed cells (69%) (Figure 6b). A significant effect was observed at the highest trolox concentration in MDA-MB-231 cells. Trolox demonstrated significant effects in inhibiting the antiproliferative activity induced by ESE-one in both cell lines suggesting that peroxyl radical partially plays a role in the antiproliferative effect induced by ESE-one in tumorigenic cell lines. Mannitol, a hydroxyl radical inhibitor, was used in combination with ESE-one (0.5 µM) in order to determine if ESE-one exerted antiproliferative activity dependent on the hydroxyl radical. Mannitol co-exposure with ESE-one resulted in 67% (20 mM) and 66% (40 mM–100 mM) cell growth in MCF-7 cell lines (Figure 7a) compared to ESE-one only exposed cells which exhibited 60% cell growth. In MDA-MB-231 cells, mannitol exposure resulted in 81% (20 mM), 79% (40 mM), 82% (80 mM) and 84% (100 mM) cell growth compared to cells exposed to ESE-one only (74%) (Figure 7b). These results demonstrated that mannitol exerted no significant effect on the growth inhibitory effect of ESE-one in both MCF-7 and MDA-MB-231 cells. This suggests that hydroxyl radical does not play a role in the growth inhibitory pathway induced by ESE-one.

**Figure 8:**
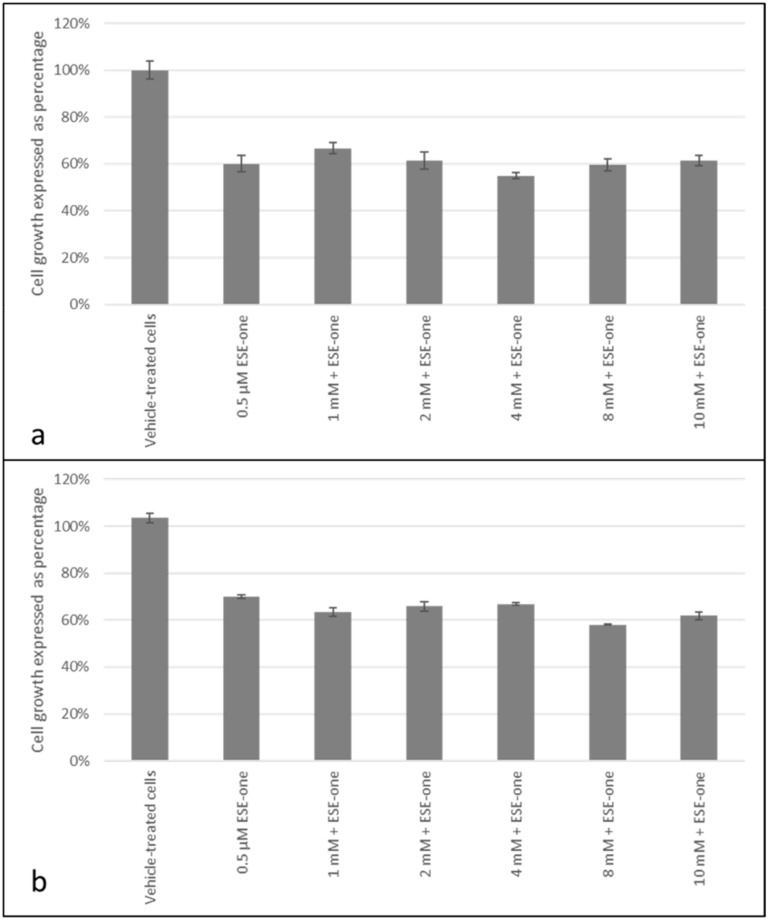
Cell growth inhibition graphs of MCF-7 and MDA-MB-231 cells exposed to ESE-one in combination with sodium azide (oxygen singlet inhibitor). Sodium azide exposure to MCF-7 and MDA-MB-231 cells did not oppose the antiproliferative effect of ESE-one in both cell lines. (**a**) MCF-7 cells, (**b**) MDA-MB-231 cells. No significant differences were observed in cell growth when compared to ESE-one treated cells (*p* > 0.05).

Cells were exposed to ESE-one in the presence and absence of sodium azide (1mM to 10 mM), an inhibitor of singlet oxygen, to determine if the antiproliferative activity exerted by ESE-one is dependent on singlet oxygen. Co-exposure to sodium azide resulted in 67% (1 mM), 61% (2 mM), 55% (4 mM), 59% (8 mM) and 61% (10 mM) cell growth compared to 60% cell growth induced by ESE-one exposure in MCF-7 cells (Figure 8a). In MDA-MB-231 cells, sodium azide exposure demonstrated a 63% (1 mM), 66% (2 mM), 67% (4 mM), 58% (8 mM) and 62% (10 mM) cell growth compared to 70% growth induced by ESE-one only exposure (Figure 8a). Thus, no statistically significant differences were observed between ESE-one only exposed cells and cells exposed to ESE-one and sodium azide in either cell line suggesting that singlet oxygen does not play a role in the growth inhibitory pathway exerted by ESE-one.

**Figure 9:**
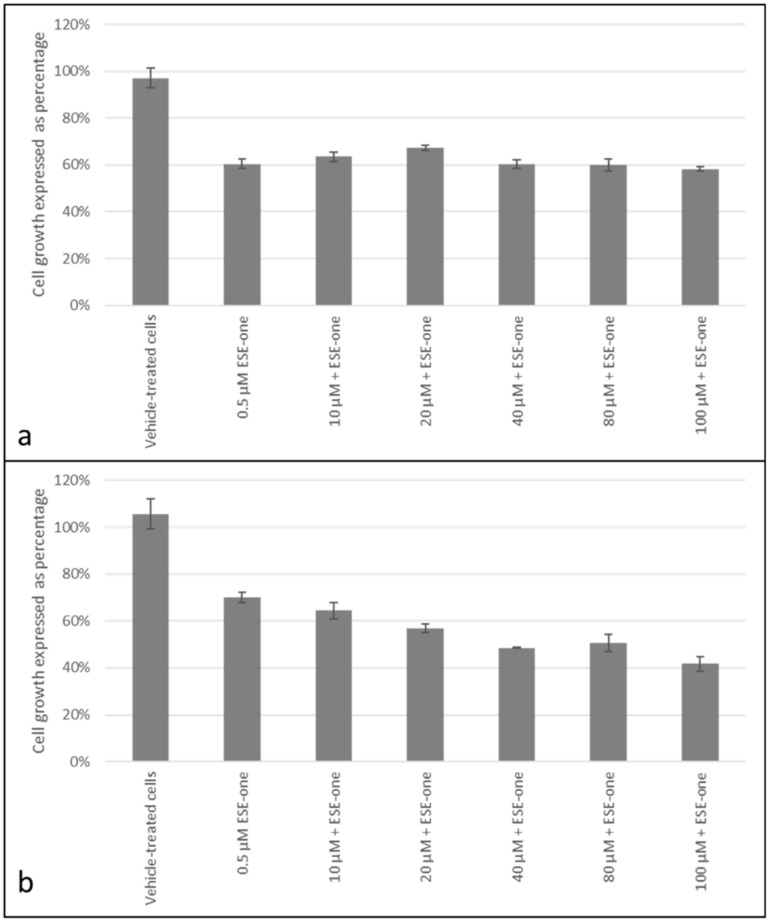
Cell growth inhibition graphs of MCF-7 and MDA-MB-231 cells exposed to ESE-one in combination with carboxy-PTIO (nitric oxide inhibitor). Carboxy-PTIO exposure to MCF-7 and MDA-MB-231 cells did not oppose the antiproliferative effect of ESE-one in both cell lines. (**a**) MCF-7 cells, (**b**) MDA-MB-231 cells. No significant differences were observed in cell growth when compared to ESE-one treated cells (*p* > 0.05).

Carboxy-PTIO, nitric oxide inhibitor, was used in combination with ESE-one (0.5 µM) in order to determine if ESE-one exerted antiproliferative activity dependent on nitric oxide. Co-exposure to carboxy-PTIO resulted in 64% (10 µM), 67% (20 µM), 60% (40 and 80 µM) and 58% (100 µM) compared to ESE-one only exposed cells which resulted in 60% cell growth in MCF-7 cells (Figure 9a). In MDA-MB-231 cells, carboxy-PTIO exposure resulted in 64% (10 µM), 57% (20 µM), 49% (40 µM), 51% (80 µM) and 42% (100 µM) cell growth compared to ESE-one only exposed cells which demonstrated a 70% cell growth (Figure 9b). Results thus indicated that carboxy-PTIO has no significant effect on the antiproliferative activity exerted by ESE-one in either cell line suggesting that cell growth inhibition is not dependent on nitric oxide.

Only three ROS inhibitors had a significant inhibitory effect on the antiproliferative activity of ESE-one, namely tiron, DMTU and trolox. Hence, subsequent experiments that investigated cell morphology were done exposing cells to ESE-one in the presence and absence of tiron (5 mM), DMTU (8 mM) and trolox (80 µM). These scavengers and doses were selected based on the above-mentioned crystal violet studies that demonstrated that the scavengers inhibited the antiproliferative activity optimally at these doses.

### 2.4. ROS Scavengers Oppose the Cell Rounding Effect of ESE-One

**Figure 10:**
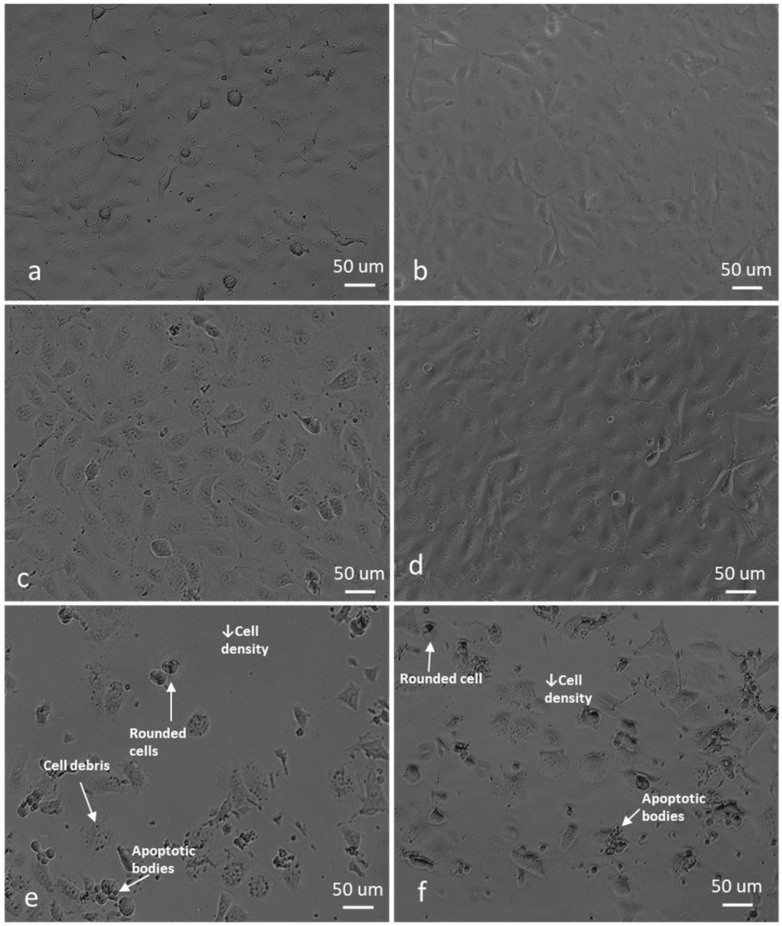
Light micrographs of MCF-7 and MDA-MB-231 cells propagated in growth medium, vehicle-treated cells and ESE-one-exposed cells. ESE-one exposed cells resulted in low cell density, rounded cells and an appearance of apoptotic bodies compared to negative control cells in both MCF-7 and MDA-MB-231 cells. (**a**) MCF-7 cells propagated in growth medium, (**b**) MDA-MB-231 cells propagated in growth medium, (**c**) MCF-7 vehicle-treated cells, (**d**) MDA-MB-231 vehicle-treated cells, (**e**) MCF-7 cells exposed to 0.5 µM ESE-one, (**f**) MDA-MB-231 cells exposed to 0.5 µM ESE-one.

**Figure 11:**
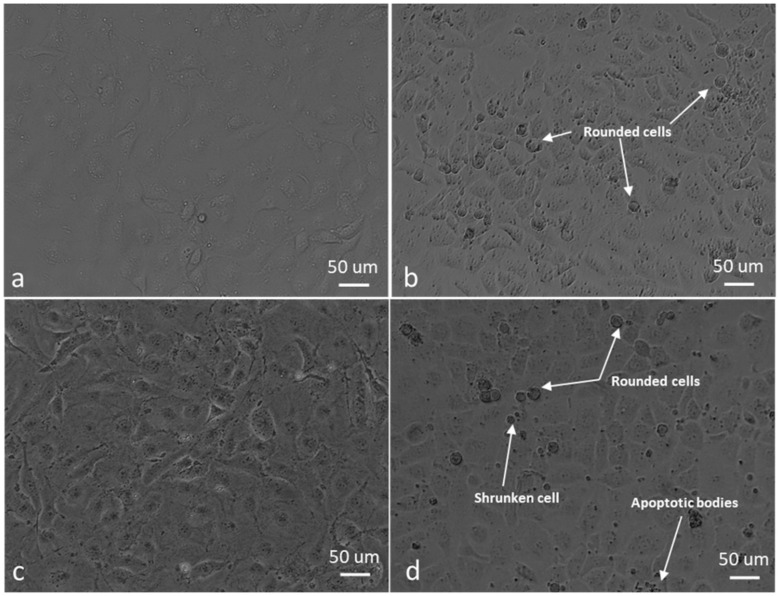
Light micrographs of MCF-7 and MDA-MB-231 cells exposed to tiron alone, and tiron in combination with ESE-one. Tiron co-exposure with ESE-one resulted in rounded cells and apoptotic bodies in both MCF-7 and MDA-MB-231 cell lines compared to cells exposed to tiron only. However, there was a decrease in rounded cells in the tiron co-exposed cells compared to ESE-one only exposed cells. (**a**) MCF-7 cells exposed to 5 mM tiron, (**b**) MDA-MB-231 cells exposed to 5 mM tiron, (**c**) MCF-7 cells exposed to tiron and ESE-one, (**d**) MDA-MB-231 cells exposed to tiron and ESE-one.

**Figure 12:**
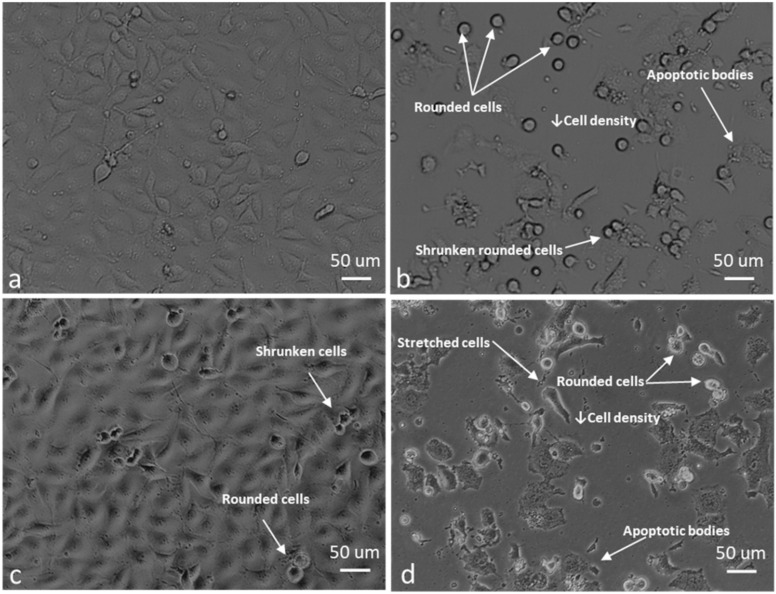
Light micrographs of MCF-7 and MDA-MB-231 cells exposed to DMTU alone, and DMTU in combination with ESE-one. DMTU co-exposure with ESE-one resulted in rounded cells, shrunken cells, stretched cells and apoptotic bodies in both MCF-7 and MDA-MB-231 cell lines compared to cells exposed to DMTU only. However, there was a decrease in rounded cells in the DMTU co-exposed cells compared to ESE-one only exposed cells. (**a**) MCF-7 cells exposed to 8 mM DMTU, (**b**) MDA-MB-231 cells exposed to 8 mM DMTU, (**c**) MCF-7 cells exposed to DMTU and ESE-one, (**d**) MDA-MB-231 cells exposed to DMTU and ESE-one.

**Figure 13:**
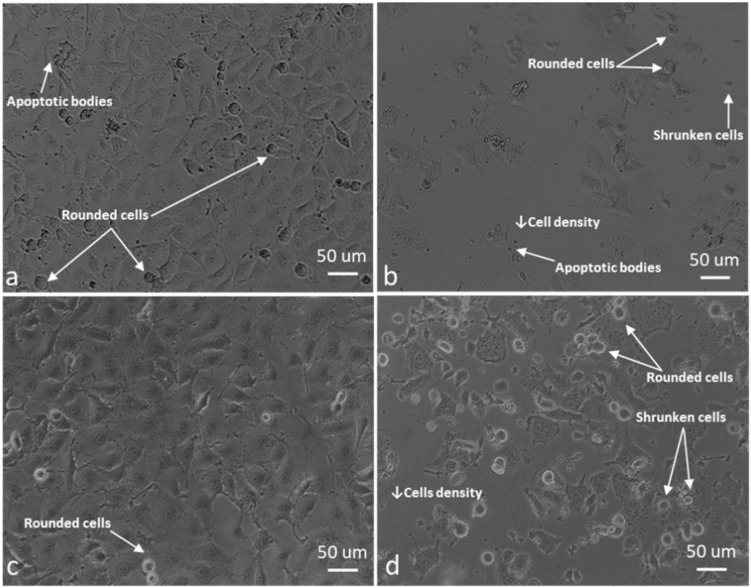
Light micrographs of MCF-7 and MDA-MB-231 cells exposed to trolox alone and DA-MB-231 cell lines compared to cells exposed to trolox only. However, there was a decrease in rounded cells in the trolox co-exposed cells compared to ESE-one only exposed cells. (**a**) MCF-7 cells exposed to 80 µM trolox, (**b**) MDA-MB-231 cells exposed to 80 µM trolox, (**c**) MCF-7 cells exposed to trolox and ESE-one, (**d**) MDA-MB-231 cells exposed to trolox and ESE-one.

**Figure 14:**
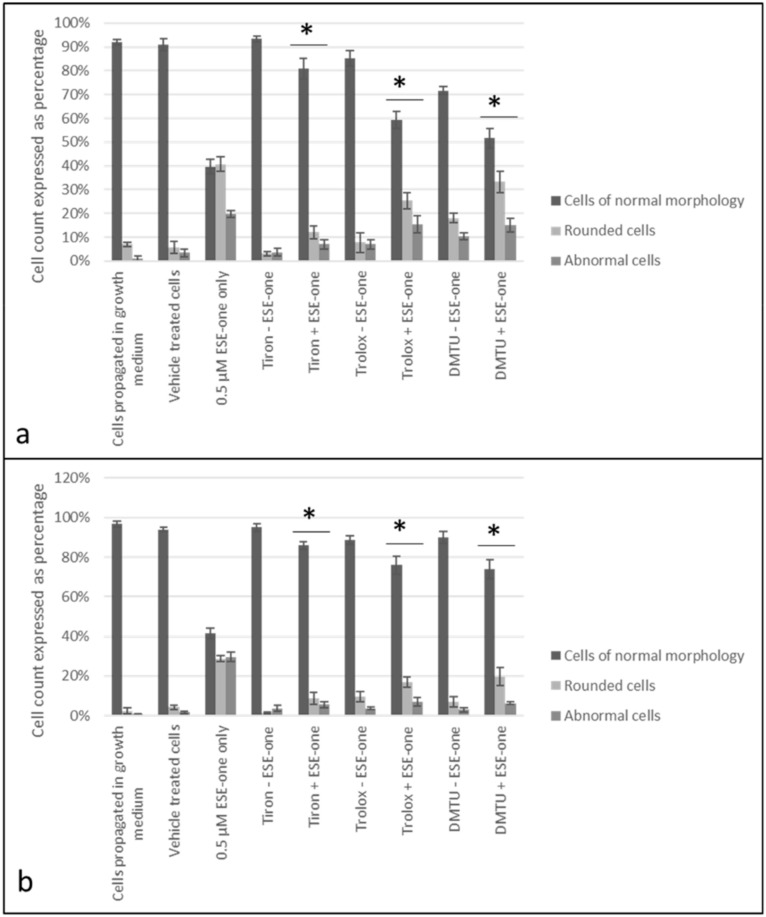
Quantification of light microscopy photographs of MCF-7 and MDA-MB-231 cells exposed to ESE-one in the presence or absence of ROS inhibitors (tiron, DMTU and trolox). ESE-one combination exposure with tiron, DMTU and trolox resulted in fewer rounded cells, fewer abnormal cells and more normal cells compared to ESE-one only exposed cells in MCF-7 and MDA-MB-231 cells. Tiron co-exposure demonstrated the most rescue effects in both MCF-7 and MDA-MB-231 cell lines with 81% and 86% normal cells respectively. (**a**) MCF-7 cells, (**b**) MDA-MB-231 cells. An asterisk (*) indicates *p*-value (*p* < 0.05) compared to ESE-one treated cells.

For morphology studies, cells were exposed to 0.5 µM ESE-one in the absence and presence of tiron (5 mM), DMTU (8 mM) and trolox (80 µM) for 24 h since the proliferation studies showed partial or complete inhibition of the antiproliferative activity exerted by ESE-one by the three aforementioned ROS inhibitors. Thereafter, light microscopy images were captured to assess the change in cell morphology when exposed to the ESE-one in comparison with cells exposed to both ESE-one and ROS inhibitors (tiron, DMTU and trolox) (Figure 10, Figure 11, Figure 12, Figure 13 and Figure 14).

ESE-one exposure further resulted in decreased cell density, shrunken cells, blebbing and appearance of apoptotic bodies in both MCF-7 and MDA-MB-231 cells. Exposure to only ESE-one resulted in 40% rounded cells and 20% abnormal cells (cells that are stretched and/or shrunken or demonstrating blebbing/apoptotic bodies) respectively in MCF-7 cells (Figure 10e and Figure 14) whereas MDA-MB-231 cells demonstrated 29% rounded and 30% abnormal cells (Figure 10f and Figure 14).

Combination exposure with tiron and ESE-one resulted in 12% rounded cells and 9% abnormal cells in MCF-7 cells, respectively (Figure 10c and Figure 14) and only 9% rounded cells and 4% abnormal cells in MDA-MB-231 cells, respectively (Figure 11d and Figure 14). DMTU co-exposure resulted in an appearance of rounded cells (33% and 20%) and abnormal cell morphology (15% and 6%) in MCF-7 (Figure 12c and Figure 14) and MDA-MB-231 (Figure 12d and Figure 14) cells, respectively. Trolox co-exposure with ESE-one also demonstrated fewer rounded cells compared to ESE-one only exposure at 25% and 17% rounded cells in MCF-7 (Figure 13c and Figure 14) and MDA-MB-231 cells (Figure 13d and Figure 14), respectively. Trolox co-exposure also resulted in less abnormal cells in MCF-7 (15%) and MDA-MB-231 (7%) cells compared to ESE-one only exposed cells.

Morphology studies suggest that all three ROS inhibitors oppose the effects of ESE-one in MCF-7 (Figure 14a) and MDA-MB-231 (Figure 14b) cells. This is observed in the cell morphology micrographs where ESE-one only exposed cells have significantly low cell density, increased cell rounding, shrunken cells and apoptotic bodies whereas these effects are at a lesser extent in tiron-, DMTU- and trolox co-exposure in both cell lines. Tiron had the most prominent inhibitory effect on the activity exerted by ESE-one compared to trolox and DMTU as the co-exposure demonstrated less cell rounding and more normal cells (81% and 86%) compared to ESE-one alone suggesting that tiron exposure can potentially rescue the cells from the antiproliferative and antimitotic effect of ESE-one in both MCF-7 and MDA-MB-231 cells. This suggests that superoxide anion, hydrogen peroxide and peroxyl radical play a role in the antimitotic effects exerted by ESE-one, however, superoxide anion has the most prominent role.

## 3. Discussion

Moderate elevated ROS are associated with various pathologies including cancer; however, excessive quantities of ROS including hydrogen peroxide and superoxide anions are cytotoxic [14,15]. ROS was reported to induce apoptotic cell death in MCF-7 and MB-MDA--231 breast cancer cell lines which was attenuated by NAC indicating the role of antioxidants in oxidative stress [16]. Fluorescent microscopy studies by means of DCFDA and DHE indicated that sulphamoylated compounds induce a higher fluorescent intensity compared to non-sulphamoylated compounds, observed when ESE-15-ol, ESE-one and ESE-ol induced a greater fluorescent intensity compared to their non-sulphamoylated counterparts (EE-15-ol, EE-one and 2E-diol) in MCF-7 and MDA-MB-231 cells. However, this effect was observed more prominently in MCF-7 cells and to a lesser extent in MDA-MB-231 cells. The high green and red fluorescent intensity observed in cells exposed to sulphamoylated compounds compared to cells exposed to the non-sulphamoylated counterparts suggests that the sulphamoylated compounds induced hydrogen peroxide and superoxide anion production [17]. It was previously reported that 2-methoxyestradiol-bis-sulphamate, another sulphamoylated estradiol compound, induced ROS (hydrogen peroxide and superoxide anion) production in MCF-7 cells which is similar to data obtained in the current study using other in silico designed estradiol sulphamoylated compounds [1]. Due to the cytotoxic effects of high ROS, proliferation studies were conducted to assess the role of ROS on the antiproliferative effects of sulphamoylated estradiol compounds.

Cell proliferation studies demonstrated a statistically significant decrease in cell proliferation after exposure to the sulphamoylated compounds compared to exposure to the non-sulphamoylated compounds. This effect was more prominent in MCF-7 cells indicating that the ER positive adenocarcinoma cells are more sensitive to the antiproliferative effects exerted by sulphamoylated compounds compared to ER negative adenocarcinoma cells (MDA-MB-231). Thus, decreased proliferation correlated with increased hydrogen peroxide and superoxide production after exposure to the sulphamoylated compounds. This effect was confirmed by the literature where a sulphamoylated compound, ESE-ol (also known as C16), significantly inhibited cell growth in MCF-7 and MDA-MB-231 breast tumorigenic cells after 24 h exposure (200 nM) [2]. Spectrophotometry data confirmed the fluorescent microscopy results indicating that ROS are responsible for the antiproliferative effects of sulphamoylated estradiol compounds. However, no studies to date have demonstrated the exact mechanism of action utilized by these sulphamoylated compounds to induce increased ROS generation. The major contributor for ROS production in actively proliferating cells is mitochondrial generation of superoxide and subsequent hydrogen peroxide production [18]. Superoxide, which is made by receiving an electron from nicotinamide adenine dinucleotide phosphate, is converted to hydrogen peroxide via superoxide dismutase (SOD), and catalase converts hydrogen peroxide to water. ROS are generated via the mitochondria as a typical cell maintenance mechanism whereby a balance is maintained between ROS generation and ROS elimination [19]. Furthermore, ROS are mainly produced by the mitochondria thus aberrant mitochondrial function is frequently associated with inconsistent ROS quantities. In addition, mitochondria are also the main target of ROS [20]. An elevation in ROS leads to an aberrant change in the mitochondrial membrane potential resulting in membrane depolarization and subsequent apoptosis induction [21,22,23]. Future studies will involve instigating the cell signaling utilized by these sulphamoylated analogues resulting in the increase in ROS generation identified in this study.

Due to previous studies that demonstrated that sulphamoylated compounds induce antiproliferative effects via ROS production [8], ROS scavengers were utilized to identify the different ROS involved in the antiproliferative activity exerted by sulphamoylated compounds such as ESE-one. Three out of six scavengers (tiron, trolox, DMTU) had a rescue effect in the antiproliferative action induced by ESE-one. The cell proliferation results suggest that superoxide anion, peroxyl radical and hydrogen peroxide are involved in the cell death effect induced by ESE-one in breast tumorigenic cell lines as tiron, trolox and DMTU had an opposing effect on ESE-one in both cell lines. Homobrassinin, an anticancer indole phytoalexin compound, induced intracellular ROS production in human colorectal cancer cells (Caco2) which was associated with apoptosis induction and decreased cell density; however, trolox significantly decreased the ROS intensity [24]. Furthermore, a bacterial cyclic lipopeptide with anticancer properties (surfactin) induced apoptotic cell death in MCF-7 cells via ROS production which was inhibited by NAC and catalase [25]. This is similar to the data obtained from the current study where co-exposure with ROS inhibitors opposed the antiproliferative effect of ESE-one. The effect of ROS scavengers on the antiproliferative effect of sulphamoylated estradiol compounds has not yet been reported.

The current study demonstrated that ESE-one exposure resulted in decreased cell density, shrunken cells, blebbing and appearance of apoptotic bodies. Other sulphamoylated estradiol compounds like ESE-ol and EMBS are reported to possess antimitotic effects which are evident in the exposed cells experiencing a metaphase arrest manifested in rounded cells [2,26]. Previous studies also demonstrated that another sulphamoylated estradiol analogue (ESE-ol) induced cell rounding and loss of cell density and apoptotic characteristics which was observed in MCF-7 and MDA-MB-231 cells [2]. In the current study, morphology indicated that the cell rounding effect induced by ESE-one was partially reversed by tiron, trolox and DMTU as cells co-exposed with ESE-one and the three ROS inhibitors demonstrated fewer rounded cells and more non-rounded cells. This effect was observed in both MCF-7 and MDA-MB-231 cells, however the effect was more prominently observed in the MCF-7 cells. Tiron opposed ESE-one’s antimitotic effect to a greater extent suggesting that ESE-one induces cell rounding via the superoxide pathway. Co-exposure with ROS inhibitors depicted the specific ROS involved in the cell rounding effect of ESE-one; this combination has not been reported to date.

This in vitro study demonstrated that ESE-one is an antiproliferative and antimitotic compound in breast tumorigenic cells (MCF-7 and MDA-MB-231) which operates via ROS production. Furthermore, the current study indicated that superoxide anion, peroxyl radical and hydrogen peroxide are the specific ROS utilized in the mechanism of action induced by ESE-one resulting in decreased cell proliferation and an increase in rounded cells.

## 4. Materials and Methods

### 4.1. Cell Lines

MDA-MB-231 is a triple negative tumorigenic breast cell line derived from a human adenocarcinoma metastatic site [27]. These cells do not express estrogen receptor (ER), progesterone receptor (PR) and human epidermal growth factor receptor 2 (HER2). The MCF-7 cell line is an adenocarcinoma ER positive, PR positive and HER2 negative breast epithelial cell line [1]. MCF-7 and MDA-MB-231 cell lines were obtained from the American Type Culture Collection (American Type Culture Collection, Manassas, VA, USA). The MDA-MB-231 and the MCF-7 cell lines were cultured in 25 cm^2^ tissue flask in Dulbecco’s minimum essential medium eagle (DMEM) with 10% heat-inactivated fetal calf serum (FCS) (56 °C, 30 min), 100 U/mL penicillin G, 100 mg/mL streptomycin and fungizone (250 mg/L) at 37 °C and 5% CO_2_.

### 4.2. Reagents

All reagents were obtained from Sigma Chemical Co (Sigma Chemical Co., St. Louis, MO, USA) unless otherwise specified. DCFDA, HE and ROS inhibitors were manufactured and obtained from Sigma Chemical Co. (Sigma Chemical Co., St. Louis, MO, USA). Crystal violet dye was manufactured and provided by Merck & Co., Inc. (Merck & Co., Inc., Kenilworth, NJ, USA).

### 4.3. Methods

#### 4.3.1. Hydrogen Peroxide and Superoxide Production (Fluorescent Microscopy)

The effects of the sulphamoylated and non-sulphamoylated estradiol compounds on hydrogen peroxide production were quantified as an indicator of oxidative stress. DCFDA was used to measure hydrogen peroxide production. DCFDA, a non-fluorescent probe, is oxidized to its fluorescent derivative DCF by hydrogen peroxide [28,29].

MCF-7 and MDA-MB-231 cells were seeded in 24-well plates at a density of 20,000 cells per well and incubated for 24 h at 37 °C and 5% CO_2_ to allow for attachment. Cells were exposed to various sulphamoylated compounds (0.5 µM) (ESE-15-ol, ESE-one and ESE-ol) and non-sulphamoylated compounds (0.5 µM) (EE-one, EE-15-ol and 2-E-diol) for 24 h at 37 °C and 5% CO_2_. Upon termination, cells were washed with phosphate buffer solution (PBS). Cells were then incubated with 20 μM DCFDA for 25 min at 37 °C and 5% CO_2_. Samples were washed with PBS and 0.5 µL PBS was added to each well. Zeiss Axiovert CFL40 microscope, Zeiss Axiovert MRm monochrome camera (Zeiss, Oberkochen, Baden-Württemberg, Germany) and Zeiss filter 9 were employed to capture images of DCFDA-stained (green) cells. Fluorescence images were analyzed using Image J software (National Institutes of Health, Bethesda, MD, USA). The fluorescent intensity of at least 100 cells was evaluated per condition in each experiment.

The effects of the sulphamoylated and non-sulphamoylated estradiol compounds on superoxide production were quantified as an indicator of oxidative stress. DHE was used to measure superoxide production. Superoxide oxidizes DHE to form a fluorescent red 2-hydroethidine cation [30]. MCF-7 and MDA-MB-231 cells were seeded in 24-well plates at a density of 20,000 cells per well and incubated for 24 h at 37 °C and 5% CO_2_ to allow for attachment. Cells were then exposed to sulphamoylated compounds (0.5 µM) (ESE-15-ol, ESE-one and ESE-ol) and non-sulphamoylated compounds (0.5 µM) (EE-one, EE-15-ol and 2-E-diol) for 24 h at 37 °C and 5% CO_2_. Upon termination, cells were washed with PBS. Cells were incubated with 10 μM DHE for 45 min at 37 °C and 5% CO_2_. Samples were washed with PBS and 0.5 µL PBS was added to each well. Zeiss Axiovert CFL40 microscope, Zeiss Axiovert MRm monochrome camera (Zeiss, Oberkochen, Baden-Württemberg, Germany) and Zeiss filter 15 were employed to capture images of DHE-stained (red) cells. Fluorescence images were analyzed using Image J software (National Institutes of Health, Bethesda, MD, USA). The fluorescent intensity of at least 100 cells was evaluated per condition in each experiment.

#### 4.3.2. Cell Proliferation (Spectrophotometry)

Crystal violet staining was used to determine the influence of ESE-one on cell proliferation in the presence and absence of ROS inhibitors. In addition, crystal violet staining was also done to determine if there is a significant differential effect exerted by sulphamoylated compounds when compared to their non-sulphamoylated compound counterparts. The crystal violet technique involves staining of the nuclei and cellular DNA with a triphenylmethane cation dye that binds to proliferating cells. Spectrophotometry was used together with crystal violet staining to obtain the absorbance of the solubilized dye at a wavelength of 570 nm [31].

MCF-7 and MDA-MB-231 cells were seeded in 96-well plates at 4000 cells per well and incubated at 37 °C and 5% CO_2_ for 24 h to allow for the attachment of cells to the plate. Cells were then exposed to 0.5 µM ESE-one since previous studies conducted in our laboratory with several sulphamoylated compounds demonstrated optimal antiproliferative activity at this dose in several tumorigenic cell lines [3]. In addition, cells were also exposed to additional sulphamoylated compounds (0.5 µM) for comparison with their non-sulphamoylated compound (0.5 µM) counterparts. Cells were exposed to ESE-one in the absence or presence of ROS scavengers (mannitol, trolox, tiron, Carboxy-PTIO, sodium azide and *N*,*N*′-dimethylthiourea (DMTU)) (Table 2) and incubated for 24 h at 37 °C and 5% CO_2_. Upon termination, cells were fixed with 1% gluteraldehyde (100 μL) at room temperature for 15 min. Gluteraldehyde was then replaced with 0.1% crystal violet (100 μL) at room temperature for 30 min. Plates were left to dry overnight. Thereafter, 0.2% triton X-100 (200 μL) was added to the plates and incubated overnight to solubilize the crystal violet. Absorbances were read by means of an EPOCH Microplate Reader (Biotek Instruments, Inc., Winooski, VT, USA)) at a wavelength of 570 nm. Data were analyzed using Microsoft Excel 2010 (Microsoft Corporation, Redmond, WA, USA).

#### 4.3.3. Cell Morphology

Light microscopy was used to investigate if the cell rounding effects induced by ESE-one are dependent on ROS formation. MCF-7 and MDA-MB-231 cells were seeded in 24-well plates at a density of 20,000 cells per well and incubated at 37 °C and 5% CO_2_ for 24 h to allow for the attachment of cells to the plate. After 24 h, cells were exposed to 0.5 μM ESE-one in the presence and absence of ROS scavengers for 24 h at 37 °C and 5% CO_2_. Thereafter, an Olympus CKX53 inverted microscope (Olympus Corporation, Shinjuku City, Tokyo, Japan) was used to capture images in order to compare the morphology of cells exposed to ESE-one in presence and absence of ROS scavengers. Light microscopy images were analyzed using Image J software (National Institutes of Health, Bethesda, MD, USA). At least 100 cells were counted per condition in each experiment.

## 5. Statistics

Quantitative data were obtained from spectrophotometry (cell proliferation), fluorescent microscopy (ROS production) and light microscopy (morphology). Qualitative data were obtained from light microscopy and fluorescent microscopy. Three independent experiments were conducted where the average and the standard deviation were calculated. Averages are illustrated by bar charts and standard deviations are shown with error bars. A *p*-value < 0.05 calculated by means of Student’s *t*-test was used for statistical significance and is indicated by an asterisk (*). Fluorescent and light microscopy images were analyzed using Image J software (National Institutes of Health (Bethesda, MD, USA). The fluorescent intensity of at least 100 cells was evaluated per condition in each experiment and at least 100 cells were counted in the light microscopy images per condition in each experiment.

## 6. Conclusions

Data from the current study revealed that ESE-one induced ROS production, rounded cells and apoptotic bodies and reduced cell growth in breast tumorigenic cell lines. These effects can be inhibited by ROS (tiron, trolox and DMTU) inhibitors indicating that superoxide anion, peroxyl radical and hydrogen peroxide are essential for the pathways induced by ESE-one. This study is the first to report that superoxide anion, peroxyl radical and hydrogen peroxide are required for ESE-one-induced effects including decreased cell proliferation and increased cell rounding in breast tumorigenic cell lines. This research contributes towards future mechanistic and pharmacogenomic studies including the molecular mechanisms needed for targeting specific ROS to inhibit cell growth in cancer cells and thereby improve current therapy targeting ROS-induced pathways in cancer to ultimately and selectively kill cancer cells.

## Figures and Tables

**Table 1:** In silico designed sulphamoylated and non-sulphamoylated compounds. Structures were created by MH Visagie using ACD/ChemSketch version 1101 released on 2007/10/19 (Advanced Chemistry Development, Inc., ACD/Labs, Toronto, ON, Canada). Chemical names were verified using ChemSpider (released in 2008, Royal Society of Chemistry, Raleigh, NC, USA).

Non-Sulphamoylated Compound	Sulphamoylated Compound
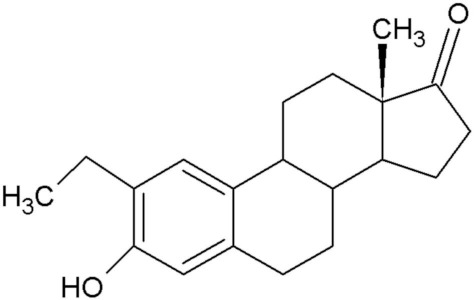 2-Ethyl-3-hydroxyestra-1(10),2,4-trien-17-one (EE-one) also known as C5).	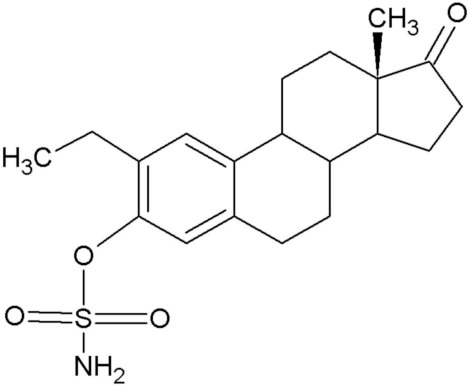 2-Ethyl-17-oxoestra-1,3,5(10)-trien-3-yl sulphamate (ESE-one) also known as C15.
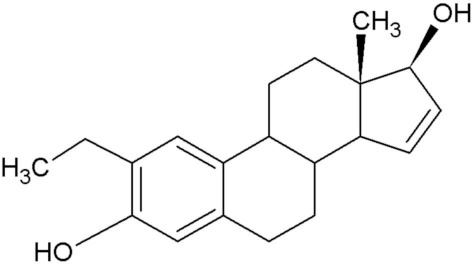 2-Vinylestra-1(10),2,4,16-tetraene-3,17-diol (EE-15-ol) also known as C11.	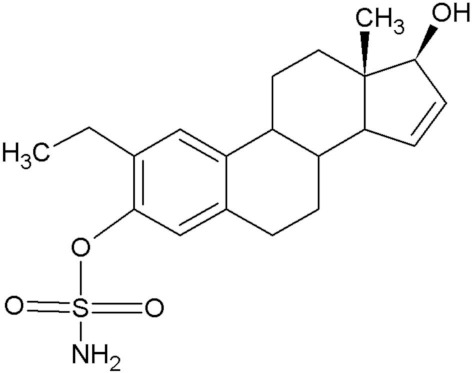 2-Ethyl-17-hydroxyestra-1(10),2,4,16-tetreane-3-yl sulfamate (ESE-15-ol) also known as C10.
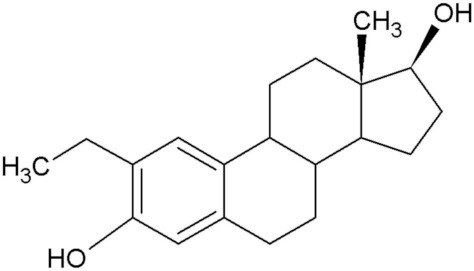 2-Ethylestra-1(10),2,4-triene-3,17-diol (2-E-diol) also known as C13.	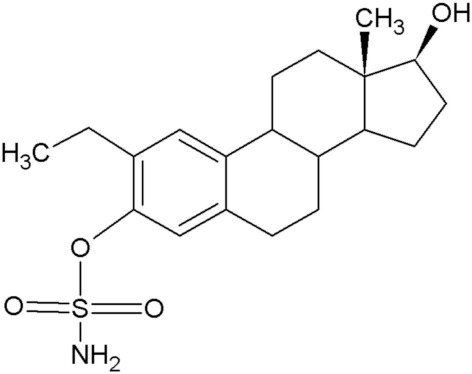 2-Ethyl-17-hydroxyestra-1(10),2,4-trien-3-yl sulfamate (ESE-ol) also known as C16.

**Table 2:** ROS scavengers and concentration ranges that were used.

Reactive Oxygen Species	Scavenger	Concentration
Hydrogen peroxide	DMTU	1–10 mM [32]
Hydroxyl radical	Mannitol	20–100 mM [32]
Nitric oxide	Carboxy-PTIO	10–100 µM [32,33]
Peroxyl radical	Trolox	10–100 µM [34,35]
Singlet oxygen	Sodium azide	1–10 mM [32,36])
Superoxide anion	Tiron	1–10 mM [37,38]
